# Brood‐tending males in a biparental fish suffer high paternity losses but rarely cuckold

**DOI:** 10.1111/mec.14857

**Published:** 2018-09-27

**Authors:** Aneesh P. H. Bose, Holger Zimmermann, Jonathan M. Henshaw, Karoline Fritzsche, Kristina M. Sefc

**Affiliations:** ^1^ Institute of Biology University of Graz Graz Austria

**Keywords:** alternative reproductive tactics, cichlid, Lake Tanganyika, microsatellite genotyping, social monogamy, *Variabilichromis moorii*

## Abstract

Extra‐pair paternity within socially monogamous mating systems is well studied in birds and mammals but rather neglected in other animal taxa. In fishes, social monogamy has evolved several times but few studies have investigated the extent to which pair‐bonded male fish lose fertilizations to cuckolders and gain extra‐pair fertilizations themselves. We address this gap and present genetic paternity data collected from a wild population of *Variabilichromis moorii*, a socially monogamous African cichlid with biparental care of offspring. We show that brood‐tending, pair‐bonded males suffer exceptionally high paternity losses, siring only 63% of the offspring produced by their female partners on average. The number of cuckolders per brood ranged up to nine and yet, surprisingly, brood‐tending males in the population were rarely the culprits. Brood‐tending males sired very few extra‐pair offspring, despite breeding in close proximity to one another. While unpaired males were largely responsible for the cuckoldry, pair‐bonded males still enjoyed higher fertilization success than individual unpaired males. We discuss these results in the context of ecological and phenotypic constraints on cuckoldry and the fitness payoffs of alternative male tactics. Our study provides new insights into how pair‐bonded males handle the trade‐off between securing within‐pair and extra‐pair reproduction.

## 1 INTRODUCTION

High rates of extra‐pair matings leading to appreciable paternity losses are commonplace in many socially monogamous species (Cohas & Allainé, 2009; Griffith, Owens, & Thuman, 2002). In species where pair‐bonded males also provide parental care, cuckoldry diminishes the fitness benefits of male care due to lower average relatedness between males and their broods. Cuckoldry is therefore expected to select for traits in males that help them safeguard their paternity (such as mate guarding, territory guarding or repetitive mating with their social partner: Van Rhijn, 1991; Fishman, Stone, & Lotem, 2003; but see Kokko & Morrell, 2005). Simultaneously, high rates of extra‐pair paternity imply that cuckolding could be a viable alternative route to fitness, and pair‐bonded males may also experience selection favouring the pursuit of extra‐pair matings. What then determines the extent to which a pair‐bonded, caregiving male should seek extra‐pair matings? The level of extra‐pair paternity in a population is expected to be determined by the availability of reproductive males and how effective they are at cuckoldry, the willingness of females to mate multiply, and the capability of paired males to limit competitors’ access to their female partners (Jennions & Petrie, 2000). Overall, the question of how extra‐pair paternity arises in socially monogamous species has received considerable research attention in both birds (Brouwer et al., 2017; Griffith et al., 2002; Kempenaers & Schlicht, 2010) and mammals (Clutton‐Brock & Isvaran, 2006; Cohas & Allainé, 2009). According to classic theory based largely on bird and mammal systems, males cannot simultaneously maximize their within‐pair and their extra‐pair fitness, which should select for males that optimize the trade‐off between protecting their reproductive success at home and seeking extra‐pair copulations elsewhere (Hasselquist & Bensch, 1991; Kokko & Morrell, 2005). Pair‐bonded males faced with this trade‐off may compromise and employ a combination of reproductive tactics whereby they guard and mate with their female partners during their females’ fertile periods but seek extra‐pair matings outside this period (Fishman et al., 2003; e.g., northern wheatear, *Oenanthe oenanthe*, Currie, Burke, Whitney, & Thompson, 1998). How strongly paternity assurance at home trades off with extra‐pair mating success elsewhere depends to a large degree on the extent of overlap in female fertile periods within the population (Birkhead & Biggins, 1987; Canal, Jovani, & Potti, 2012; García‐Navas et al., 2015), and this overlap is considerable in some species.

To make sense of the evolutionary forces shaping social monogamy, the multiple phylogenetically independent origins of this mating system are a valuable resource. Although a focus on birds and mammals is understandable because social monogamy is widespread in these groups, the insights provided by other taxa should not be neglected (Brown, Morales, & Summers, 2010; Dillard, 2017; Liebgold, Cabe, Jaeger, & Leberg, 2006). For instance, although monogamous fishes represent a relatively small minority of all extant fish species, social monogamy occurs in at least 18 fish families (Whiteman & Côté, 2004; Kvarnemo, 2018; e.g., Barlow, 1991; Kvarnemo, Moore, Jones, Nelson, & Avise, 2000; Whiteman & Côté, 2003) and reports of monogamy in fishes have been increasing. Still, the topic of paternity loss has been very scantly explored in socially monogamous fishes (Coleman & Jones, 2011).

Several key differences in reproductive behaviour, parental care and other life history traits limit the transferability of insights from socially monogamous bird and mammal systems to socially monogamous fishes and vice versa. First, the trade‐off that males experience between guarding paternity and seeking extra‐pair matings may be weak in many socially monogamous fishes. This is because eggs are often fertilized externally and spawning presents a relatively short time window during which paternity must be defended (in comparison to the longer, and sometimes less conspicuous, female fertile periods in most birds and mammals). Unless female spawning is especially synchronous, this time window is unlikely to overlap considerably with other nearby spawning events, providing pair‐bonded males with more time to seek extra‐pair matings. Second, cuckoldry tactics can be particularly profitable in fishes with external fertilization and large brood sizes, affording numerous males the chance to gain paternity shares (Knapp & Neff, 2008; Taborsky, 2001). Third, body size heavily dictates the relative payoffs of territory defence versus cuckoldry in many taxa (Taborsky & Brockmann, 2010), and in fishes, indeterminate growth can lead to high variance in adult male body sizes. Fourth, most fishes do not provision their young after hatching and parental care is limited to brood defence and hygiene (Smith & Wootton, 1995). If the female can perform these parental tasks alone, then any reductions in male parental investment, or indeed any male absences from the nest, may incur lower fitness costs relative to systems (e.g., most birds) where high investment from both parents is crucial for offspring survival. Fifth, while extra‐pair copulations are largely under female control in many bird species (Birkhead & Møller, 1993), in fishes, cuckoldry typically occurs due to male intrusions with females exerting relatively less control (but see Reichard, Le Comber, & Smith, 2007; Li, Takeyama, Jordan, & Kohda, 2015; Alonzo, Stiver, & Marsh‐Rollo, 2016). These factors taken together suggest that, in fishes, pair‐bonded socially monogamous males could be highly prolific cuckolders. However, prior to our study this idea had not yet been directly investigated.

The bulk of previous research on paternity loss in fishes has focused on paternal caregiving species that do not form pair‐bonds (Cardoso et al., 2017; Cogliati, Neff, & Balshine, 2013; Coleman & Jones, 2011). This work has shown that territorial males of multiple species are indeed plastic in their reproductive behaviours, engaging in conventional tactics (e.g., courtship and resource‐holding) alongside alternative tactics (e.g., cuckoldry). However, these males must carefully weigh the benefits of seeking extra‐pair fertilizations against the costs of leaving their current offspring temporarily vulnerable (Candolin & Vlieger, 2013). For example, territorial male plainfin midshipman fish, *Porichthys notatus*, adopt both conventional and cuckolding tactics, but they cuckold primarily when they have no offspring of their own to care for (Cogliati, Balshine, & Neff, 2014; Lee & Bass, 2006). Similarly, male three‐spined sticklebacks, *Gasterosteus aculeatus*, are more likely to risk their current brood for a cuckoldry opportunity when their current brood is of low reproductive value (Candolin & Vlieger, 2013). Biparental care may partially release males from this constraint; that is, the female's presence may allow a male to seek extra‐pair fertilizations without leaving their offspring completely vulnerable. However, of the few studies that have investigated extra‐pair paternity in biparental, socially monogamous fishes, most have focused on how much paternity is lost by pair‐bonded males (e.g., *Micropterus salmoides*, DeWoody, Fletcher, Wilkins, Nelson, & Avise, 2000; *Eretmodus cyanostictus*, Taylor, Morley, Rico, & Balshine, 2003; *Telmatochromis temporalis*, Katoh, Munehara, & Kohda, 2005; *Neolamprologus pulcher*, Dierkes, Taborsky, & Achmann, 2008; *Xenotilapia rotundiventralis*, Takahashi, Ochi, Kohda, & Hori, 2011; *Pelvicachromis taeniatus*, Langen, Thünken, & Bakker, 2013; *Neolamprologus caudopunctatus*, Schaedelin, van Dongen, & Wagner, 2015) and virtually none have examined the extent to which pair‐bonded males act as cuckolders themselves.

In this study, we used *Variabilichromis moorii*, a species of socially monogamous cichlid endemic to Lake Tanganyika, Zambia. Male–female pairs jointly defend non‐overlapping rocky territories that they use for foraging and brood care (Karino, 1997, 1998; Ota, Hori, & Kohda, 2012; Sturmbauer et al., 2008). Territories can be densely clustered together in the wild (Karino, 1998; Sturmbauer et al., 2008), which might facilitate cuckoldry among brood‐tending, paired males. A previous study by Sefc, Mattersdorfer, Sturmbauer, and Koblmüller (2008) used microsatellite genotyping to reveal the extent of multiple paternity in this species, showing that the number of genetic sires per brood ranged from two to over 10 males. However, a major limitation of this previous study was that the parental genotypes were unknown, thus making it impossible to ascertain what the caregiving males’ true paternity values were or whether they gained any fertilizations outside of their pair‐bonds. Nevertheless, the findings of Sefc et al. (2008) naturally raise questions that may be posed in any mating system involving cuckoldry, namely how do brood‐tending males maximize their reproductive success in the face of rampant cuckoldry, and which individuals are gaining the extra‐pair reproductive success? Here, we use genetic paternity data from two sampling excursions to track male fertilizations in the field and answer the following questions. First, how much paternity do brood‐tending (i.e., pair‐bonded) males lose due to cuckoldry from other males? Second, do brood‐tending males also employ cuckoldry tactics, and by doing so can they fully offset their paternity losses at home? Third, how does the reproductive output of *unpaired* males compare with that of brood‐tending males? Fourth, are all unpaired males also reproductively capable, or do they comprise a mixture of reproductive and non‐reproductive individuals?

## 2 METHODS

### 2.1 Study species

*Variabilichromis moorii* is a small herbivorous cichlid found in the shallow, rocky shores of Lake Tanganyika. Adult males and females form pair‐bonds and jointly defend territories ranging in size from 1 to 4 m^2^, which can reach densities of up to 0.33 territories per square metre (Karino, 1998; Sturmbauer et al., 2008). Spawning occurs throughout the year with no discrete breeding season, though egg deposition is temporally concentrated during the week leading up to each full moon (Rossiter, 1991). The central rocks on each territory are used as a nest for depositing and hiding eggs, and brood sizes can exceed 100 offspring (Karino, 1997; Sefc et al., 2008). Pairs care for one brood at a time and parental care extends for ~100 days until the fry reach independence (Rossiter, 1991). Broods are often staggered across pairs, such that while one pair is caring for fry, their neighbours can be actively spawning (Rossiter, 1991). During the parental care period, both parents participate in defence against con‐ and hetero‐specifics, including defence against fry‐eating predators (Karino, 1997). Long‐term monitoring of known breeding territories in the wild combined with repeated identification of individuals (via genotyping) has revealed that some pair‐bonds can remain stable for long periods of time encompassing multiple consecutive broods (Holger Zimmermann, Karoline Fritzsche, Jonathan M. Henshaw, unpublished data).

### 2.2 Field collections

Over the course of two field seasons, one from 22 September to 28 October 2015 (corresponding with the dry season) and one from 4 to 20 April 2016 (rainy season), we identified *V. moorii* territories each containing an active nest within a study quadrat (~100 m × ~50 m, depth range: 1.7–12.1 m) in the south of Lake Tanganyika (8°42′29.4″S, 31°07′18.0″E). In the dry season, we identified 88 territories within the quadrat, while in the rainy season, we identified 52. For most territories, we measured the distance to the nearest neighbouring territory (centre to centre, to the nearest 0.1 m) and then used a gill net to capture the male–female pair (though a small number of adults evaded capture). We sexed the adults by visual inspection of their urogenital papillae, measured them for total length (to the nearest 0.1 cm) and took a fin clip before releasing them back to their territories (*N* = 76 brood‐tending, pair‐bonded males and 88 females in the dry season, 49 brood‐tending, pair‐bonded males and 49 females in the rainy season). We also captured the fry present on a subset of these territories in each season. Because fry become more difficult to capture as they get older, we focused on capturing broods whose fry were ≤1.5 cm in length (average length was 0.9 cm, suggesting ~1 month old, Rossiter, 1991). These fry were euthanized in MS‐222 (1 g/1 L lake water). Fry and adult fin clips were stored in 99.9% ethanol. In total, we sampled 882 fry from 33 territories in the dry season, and 1,321 fry from 42 territories in the rainy season. Each adult pair and brood were sampled once per season. We additionally fin clipped a total of 58 unpaired adults (41 males, 10 females, four unknowns in the dry season, three males in the rainy season) that were opportunistically caught within the quadrat. Paired individuals can easily be discriminated from unpaired individuals by underwater observation; paired‐bonded males remain near their territory, defend their territory from other fish and show affiliative interactions with their female partner, while unpaired males swim about more freely and are aggressively driven away from territories by pair‐bonded individuals. All preserved fry were measured for body length (to the nearest mm) and then transported along with the fin clips to the laboratory for genetic parentage analyses via microsatellite genotyping. This work was carried out under a study permit issued by the government of Zambia with permission from the Fisheries Department of Zambia.

### 2.3 Microsatellite genotyping

We followed a standard Chelex protocol (Walsh, Metzger, & Higuchi, 1991) for extracting DNA from fry tissue and an ammonium acetate precipitation protocol (Sambrook, Fritsch, & Maniatis, 1989) for fin clips taken from adults. All adults and most fry were genotyped at 14 microsatellite loci (Table 1) though four broods (138 fry in total) were genotyped at nine microsatellite loci (multiplex 1 only). We used 2.5 or 4 μl Qiagen Type‐it Multiplex PCR Master Mix for the multiplex PCRs (total PCR volume was 6 or 8 μl respectively) and the following PCR program parameters: 5 min at 95°C, followed by 28 cycles with 30 s at 95°C, 90 s at 54°C and 30 s at 72°C, final extension at 60°C for 30 min. We scored allele sizes against an internal size standard (GeneScan‐500 ROX; Applied Biosystems) with an ABI 3130xl automatic sequencer (Applied Biosystems) and genemapper 3.7 software (Applied Biosystems, Vienna, Austria).

**Table 1:** Microsatellite markers used for parentage analysis

Locus	NA	*H*_*O*_	*H*_*E*_	*p*(HWE)	*p*_adj_ (HWE)	References
Multiplex 1						
Pmv17	18	0.863	0.912	0.044	0.153	Crispo, Hagen, Glenn, Geneau, and Chapman (2007)
TmoM11	16	0.833	0.849	0.077	0.217	Zardoya et al. (1996)
Pzeb3	15	0.833	0.833	0.041	0.153	Van Oppen, Rico, Deutsch, Turner, and Hewitt (1997)
UNH2075	25	0.915	0.938	0.009	0.121	Albertson, Streelman, and Kocher (2003)
Ppun21	22	0.935	0.931	0.865	0.932	Taylor et al. (2002)
Ppun9	26	0.895	0.935	0.110	0.257	Taylor et al. (2002)
Hchi59	11	0.735	0.726	0.940	0.940	Maeda et al. (2008)
Hchi94	19	0.895	0.901	0.192	0.336	Maeda et al. (2008)
UME002	10	0.742	0.695	0.234	0.364	Parker and Kornfield (1996)
Multiplex 2						
Pmv13	26	0.905	0.935	0.133	0.267	Crispo et al. (2007)
UME003	15	0.899	0.873	0.647	0.823	Parker and Kornfield (1996)
UNH908	30	0.918	0.951	0.031	0.153	Carleton et al. (2002)
Ppun5	30	0.967	0.944	0.777	0.907	Taylor et al. (2002)
Ppun20	34	0.915	0.935	0.321	0.450	Taylor et al. (2002)

Note

NA: number of alleles; *H*_*O*_: observed heterozygosity; *H*_*E*_: expected heterozygosity; *p*(HWE) and *p*_adj_ (HWE), *p*‐values and Benjamini–Hochberg adjusted *p*‐values in tests for Hardy–Weinberg equilibrium.

### 2.4 Marker polymorphism

We estimated marker polymorphism from the sampled adults. Since there was no seasonal differentiation between the population samples (*F*_ST_ = 0.001, *p* = 0.12), we pooled the dry and rainy season samples together (*N* = 306, excluding recaptures) for marker characterization in arlequin vs. 3.5.1.2 (Excoffier, Laval, & Schneider, 2005). The markers were highly polymorphic, with an average of 21.2 alleles per locus and a mean expected heterozygosity of 0.88. Exclusion probability (assuming the mother was known) calculated across loci amounted to 0.9999997 (calculated in gerud2, Jones, 2005). All loci complied with Hardy–Weinberg expectations after correcting for multiple testing. Per locus results are given in Table 1.

### 2.5 Parentage analyses and cuckolder genotype reconstructions

We based our allele frequency estimates for the parentage analyses on all adult individuals sampled each season (dry season, *N* = 219; rainy season, *N* = 98). Parentage analyses were carried out for each brood separately with the help of colony (v 2.0.6.1, Jones & Wang, 2010) to determine the paternity share of the brood‐tending male, identify migrant fry (unrelated to both parents), estimate the number of extra‐pair sires and dams per brood and assign fry to their genetic fathers. colony uses a maximum‐likelihood model to cluster offspring into full‐sib and half‐sib groups, which reflects the various sires and dams contributing to the brood. We ran colony with the genotyping error rate set to 0% but checked the output carefully for cases where a new full‐sib group was proposed despite the fry matching at many loci to another full‐sib group in the brood. Most of these cases could be resolved as genotyping errors by re‐scoring electropherograms or repeating the PCR. In the few remaining cases, fry that mismatched with a full‐sib group at only one or two loci (e.g., due to potential null alleles, mutations or unresolved genotyping errors) were assigned to that group, while fry that mismatched at >2 loci were considered a separate group (i.e., considered to have been sired by a different male).

colony has previously been noted to show a tendency for overestimating sire numbers (by splitting of full‐sib groups), if the genetic data are not sufficiently informative (Sefc & Koblmüller, 2009). Although the number and the polymorphism of the loci used in this study are exceptionally high, we noticed multiple cases in which colony presumed that there were two separate extra‐pair males, each having sired a single fry, despite the two fry sharing the same mother. Given that the two offspring would collectively possess no more than two paternal alleles per locus, we saw no compelling reason to reject the more parsimonious assumption of one shared father in these cases. We corrected the output accordingly and assigned both fry to the same full‐sib group.

After the extra‐pair fry in each brood had been assigned to full‐sib groups, we used the fry genotypes to reconstruct the genotypes of their sires (i.e., cuckolder males). To do this, we began by identifying the maternal alleles in each fry's genotype (when inherited from the brood‐tending female) and identified the remaining alleles as being inherited from the cuckolder father. However, this method did not always result in an unambiguous reconstruction of the cuckolder's genotype for each full‐sib group, especially when the full‐sib group contained only a few fry. In the following situations, ambiguities arose that we manually resolved. First, when the brood‐tending female and her fry shared the same heterozygous genotype at a locus, for example both had X/Y, it was not possible to distinguish maternal and paternal alleles, and the paternal allele was scored as having two possibilities, X or Y. Second, when a full‐sib group of fry revealed only one paternal allele at a given locus, this could mean that their father was either homozygous at this locus or that all offspring inherited the same allele from their heterozygous father. Although the latter scenario becomes more unlikely as the number of offspring in the full‐sib group increases, we scored the cuckolder's genotype in all of these cases as potentially heterozygous, that is “paternal allele observed in offspring”/“unknown allele.” Third, in situations where three or four of the brood‐tending female's fry were collectively sired by two different cuckolders, multiple potential arrangements of the fry into full‐sib groups were possible. For example, four fry, A, B, C and D, could be arranged into two full‐sib groups (A, B) and (C, D). However, a different arrangement of the fry could also be (A, C) and (B, D). Both of these arrangements are possible (given the set of paternal alleles in the fry) and yet they would imply very different sire genotypes. To resolve these situations, we extracted the paternal alleles for each fry separately and used them to construct “partial” cuckolder genotypes where only *one* allele was known for each locus. In the above example, this would lead to four possible partial cuckolder reconstructions. This procedure eliminated any risk that we would mistakenly assemble a false sire genotype. Instead, we gained partial information about the sires’ true genotypes. Importantly, we could still search for matches between these partially reconstructed genotypes and complete, or nearly complete, genotypes in our sample (i.e., those of brood‐tending males or other reconstructed cuckolders that had sired multiple fry; explained in detail below).

For each season separately, we compiled data sets consisting of the observed multi‐locus male genotypes (from fin‐clipped males) and the reconstructed multi‐locus male genotypes (of inferred cuckolders). We conducted pairwise genotype comparisons in order to identify (a) brood‐tending males that had acted as cuckolders in other territories and (b) inferred cuckolders that had cuckolded in more than one territory. The multi‐locus genotype comparisons were carried out with a custom‐made program written in Perl, since cases of partial genetic data (i.e., when only one allele at a locus could be scored) and ambiguous cuckolder genotype reconstructions (see above) had to be accounted for (details in Supporting information Appendix S1). We omitted comparisons among partial genotype reconstructions, that is where only one allele was known per locus, as these could always be “matched” with one another. We were, however, able to meaningfully compare these partial genotypes with both the complete genotypes of brood‐tending males and the complete, or nearly complete, reconstructed genotypes of cuckolders that had sired multiple fry. When running the pairwise comparisons among these multi‐locus genotypes, we allowed for a maximum of two mismatching loci to account for possible errors in genotyping or genotype reconstruction, though we also required at least eight matching loci.

We decided upon the minimum requirement of eight matching loci based on a simulation to determine the probability that a randomly generated partial genotype (i.e., one allele per locus) would match with a randomly generated complete genotype at X different loci. For each season separately, we used population allele frequencies to randomly assemble one partial and one complete 14‐locus genotype. The number of loci at which each of these genotypes matched one another was recorded and this process was repeated 10 000 times. It became increasingly improbable for the two genotypes to randomly match at higher numbers of loci. The probability that we would observe the two genotypes randomly matching at eight loci or more was 0.2%, which we deemed to be sufficiently improbable.

As a verification of these analyses, we also used cervus (Marshall, Slate, Kruuk, & Pemberton, 1998) to search for the genetic parents of all extra‐pair fry among the fin‐clipped adults in our sample. To do this, we assembled all fry that were designated as “extra‐pair” by colony into an offspring input file and then specified all genotyped males as “candidate fathers” and all genotyped females as “candidate mothers.” While cervus uses a simulation approach to account for random matches between the genotypes of offspring and candidate parents, our highly informative microsatellite data set allowed us to base our parentage inferences on allelic exclusion alone. The offspring assignments by cervus were fully consistent with the above‐described analyses of reconstructed cuckolder genotypes. Together, these analyses allowed us to quantify how many offspring were sired (a) by brood‐tending males within their pairs, (b) by brood‐tending males outside of their pairs (i.e., through cuckoldry) and (c) by cuckolder males that were not directly sampled but were inferred from the fry genotypes. It is important to note that we captured and genotyped a very high proportion of all the brood‐tending, pair‐bonded individuals in the quadrat each season. In fact, intensive surveys of the quadrat at the end of each field season found that all but two breeding pairs in the rainy season had been fin clipped. Thus, we are confident that any fry that we could not assign to known brood‐tending individuals were most likely sired by *unpaired* individuals that did not hold nesting territories within the quadrat.

### 2.6 How many of the genotyped fry were sired by brood‐tending males and by unpaired males?

All analyses were performed in the statistical software R (version 3.3.1, R Development Core Team 2016). We assigned our genotyped fry either to known brood‐tending males or to inferred unpaired males. We added together the total number of fry that each brood‐tending male sired with his own female partner as well as with other females and then compared this to the number of extra‐pair fry sired by each unpaired male (note that we accounted for cases of cuckolders siring fry across multiple territories). We fit a generalized linear model assuming a negative binomial error distribution (GLM, using the glm.nb function in the R package MASS, Venables & Ripley, 2002) to the number of fry that each male sired across all territories in the quadrat. We included “reproductive tactic” as a predictor variable, which was split into two levels, brood‐tending males and unpaired males. Because samples were obtained from two different seasons (from two sampling excursions), we also included season (dry and rainy) and its interaction with reproductive tactic as predictor variables, dropping the interaction if not significant. Next, we quantified the proportions of each female's brood that were sired by her male partner and by unpaired males. Here, we fit a generalized linear mixed effects model using a penalized quasilikelihood approach to estimate model parameters (GLMM assuming a quasibinomial error distribution, using the glmmPQL function in the R package MASS, see Bolker et al., 2009). We included reproductive tactic, season and their interaction as predictor variables. The observations in this model were also weighted by the size of each female's brood. We fit male ID and female ID as random intercepts to account for any males that sired fry in several territories, and any females that had multiple male mates.

### 2.7 Do brood‐tending males have a greater reproductive output than unpaired males?

For brood‐tending males, within‐pair reproduction can be repeated at most every 100 days, which is the approximate duration of parental care in *V. moorii* (Rossiter, 1991). Yet, males that practice cuckoldry may do so at a much faster rate. Therefore, to effectively compare the reproductive output of brood‐tending males with unpaired males, we sought to estimate the number of fry that each male tactic sired over comparable time windows. To estimate the cuckoldry success of males within the quadrat over a ~100‐day period, we would have ideally captured and genotyped *all* broods within the quadrat ranging in age from zero to 100 days old. However, due to difficulties in capturing older fry in the field, we focused on catching and genotyping relatively young broods. In both seasons, we estimated that the average age of the genotyped broods was less than 30 days and that the majority were spawned within the same lunar cycle (based on offspring growth curves in Rossiter, 1991).

We used two contrasting approaches to estimate the reproductive output of unpaired males across the entire quadrat. First, we estimated a “best‐case scenario” for unpaired males. We divided the total number of fry that each unpaired male sired by the number of territories (i.e., broods) that we genotyped in each season and then multiplied this by the total number of territories that we observed in the quadrat each season. Doing so assumes that unpaired males that sired many fry in our sampled territories also sired many fry throughout the remainder of the quadrat (and vice versa), which likely underestimates the role of stochasticity in determining the reproductive output of unpaired males. We fit a generalized linear model assuming a negative binomial error distribution, including our estimates of reproductive output as the response variable, along with reproductive tactic, season and their interaction as predictor variables, dropping the interaction if non‐significant. This approach represents a best‐case scenario for unpaired males, because it assumes that all unpaired males cuckold successfully each lunar spawning cycle.

Second, we derived maximum‐likelihood estimates for the average reproductive success of unpaired males, allowing for the possibility of unpaired males with zero reproductive success. We assumed that the sampled cuckolders in each season were drawn from a hidden cohort of *m*_*T*_ unpaired males in the quadrat. Each of these males was assumed to have a uniform probability *p* of cuckolding any particular territory. The cuckolding success of each male then follows a binomial distribution. We calculated maximum‐likelihood estimates for *m*_*T*_ and *p* using the empirical distributions of cuckolding success in our samples (details in Supporting information Appendix S1). The estimated average reproductive success of unpaired males over the whole quadrat is then given by $\hat{p}n_{T}\overset{¯}{f}$, where $\hat{p}$ is the maximum‐likelihood estimate for the per‐territory cuckolding probability, *n*_*T*_ is the total number of territories in the quadrat and $\overset{¯}{f}$ is the average number of fry sired by a cuckolder per cuckolded territory. This approach assumes that the repeatability of cuckolding success over our sampled territories is representative of the repeatability of cuckoldry over the whole quadrat. In particular, the probability that a male cuckolds two broods *A* and *B* is the same regardless of whether the broods were spawned in the same or different lunar cycles. Quantitatively similar results were obtained when cuckolding success was assumed to follow a Poisson or a negative binomial distribution (not shown).

### 2.8 How does gonadosomatic index compare between brood‐tending and unpaired males?

Between 5 and 29 April 2018 (rainy season), we collected a sample of 43 adult male *V. moorii* (21 pair‐bonded, brood‐tending males, 22 unpaired males) from the study quadrat in the south of Lake Tanganyika (8°42′29.4″S, 31°07′18.0″E). All fish were caught using gill nets while on scuba, and males were differentiated from females by visual inspection of their urogenital papilla. Brood‐tending males could be easily distinguished from unpaired males as described above (section 2.2). The males were euthanized in MS‐222 and partially dissected within 6 hr of surfacing. We removed their digestive tracts and other organs within the peritoneal cavity leaving only the testes behind. The fish were then stored at room temperature in 70% ethanol for 5 days in order to be transported back to the laboratory for accurate weighing. After 5 days of storage in 70% ethanol, each male was measured for standard length (to the nearest 0.1 cm). Their testes were removed and weighed separately, as was their eviscerated somatic body mass (each to the nearest 0.001 g, Acculab ATL‐423‐I scale). The gonadosomatic index (GSI) for each male was calculated as:

$$\text{GSI}=100*\frac{\text{Combined testes mass}\left(g\right)}{\text{Eviscerated somatic body mass}\;\left(g\right)}$$

Standard length was compared between the two male types using a linear model, while GSI was compared using a Wilcoxon rank sum test due to uneven variance between the  groups.

## 3 RESULTS

### 3.1 Brood‐tending males lose paternity to cuckolders

*Variabilichromis moorii* nesting territories were densely distributed, with average (±*SD*) nearest neighbour distances being 2.4 ± 1.8 m in the rainy season and 2.6 ± 1.6 m in the dry season. We found that rates of multiple paternity differed between the seasons, and Table 2 provides descriptive statistics for each season separately. However, for brevity we present the following descriptive statistics after pooling data from both seasons. Average brood size was 32.1 ± 21.0 fry and ranged from six to 102 fry. Brood‐tending males sired an average of 55.1 ± 33.1% of the fry present in their territories, with paternity values ranging from 0 to 100%. We could attribute this variability in paternity to three causes. First, some males were found to be related to *none* of the fry present in their territories (this occurred in seven of 75 territories). In such cases, we could not be certain whether the brood‐tending male had suffered 100% cuckoldry or whether he had been replaced by a new male since spawning. Due to this uncertainty, we conducted all the analyses in this paper twice, once including these seven territories (*N* = 75), and again after omitting them (*N* = 68). Note that our results were qualitatively similar either way, and we only present results from the analyses in which these territories were omitted. Second, in some territories we detected a proportion of the fry that was related to *neither* of the parents (this occurred in 22 of the remaining 68 territories). Females were related to 96.8 ± 7.1% of the fry present in their territories, with maternity values ranging from 63.2 to 100%. We could trace the origin of four of these unrelated fry (from three of the 22 territories) and identify their true genetic parents among our sample of *V. moorii* pairs. For clarity, we henceforth use the term “brood” to refer to the fry attributable to *just* the brood‐tending female on each territory. Third, we detected an average of 2.1 ± 2.1 extra‐pair sires per brood, ranging from zero to nine cuckolders per brood‐tending female. After omitting uncertain cases of territory takeover (i.e., cases of 0% paternity by the brood‐tending male), and accounting for fry who were living “abroad” in other territories, we calculated that brood‐tending males sired an average of 63.2 ± 30.2% of their female partners’ broods, ranging from 7.9 to 100%.

**Table 2:** Parentage by season after omitting possible territory takeover events (dry season *N* = 31; rainy season *N* = 37 pairs)

Season	Number of fry on territory	Male paternity share over fry on territory (%)	Female maternity share over fry on territory (%)
Dry	29.8 ± 20.8	73.4 ± 26.2	95.8 ± 9.1
Rainy	33.8 ± 19.3	50.3 ± 28.0	97.6 ± 4.8

	Male paternity share over female partner's brood (%)	Number of extra‐pair sires per brood	Average paternity share per extra‐pair sire (%)
Dry	76.7 ± 26.5	1.5 ± 2.0	15.7 ± 10.4
Rainy	51.9 ± 28.6	2.5 ± 2.1	18.9 ± 14.0

Note

All summary statistics are given as mean ± 1*SD*

### 3.2 Brood‐tending males rarely engage in cuckoldry

**Figure 1:**
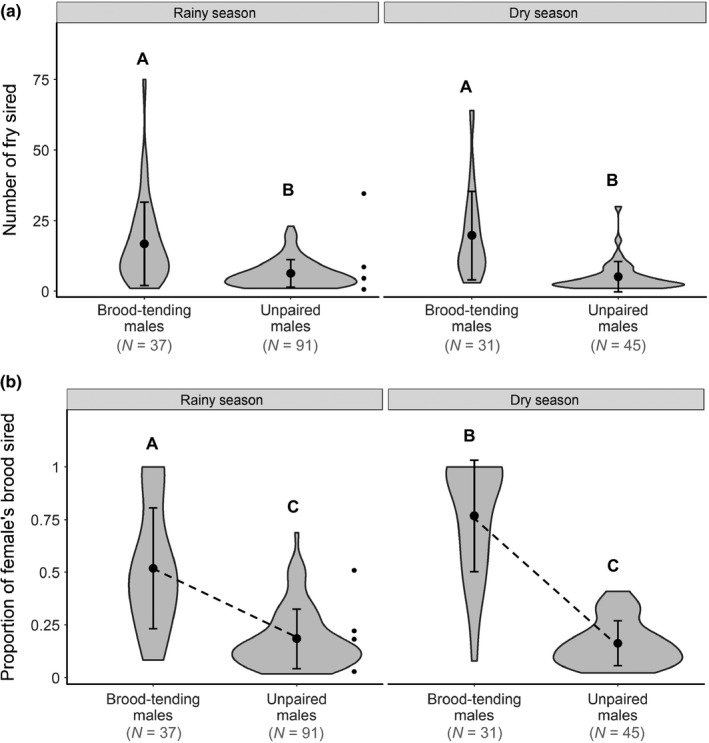
Violin plots showing the average reproductive success gained by brood‐tending males and unpaired males, compared between seasons after possible takeovers had been omitted. (a) The total number of fry sired across genotyped broods differed significantly between brood‐tending and unpaired males, independent of season. (b) Brood‐tending males sired a larger proportion of the young within their broods than unpaired males; the difference was stronger in the dry season than in the rainy season (note the different slopes of the dotted lines). Moreover, brood‐tending males sired larger proportions of each female's brood in the dry season than in the rainy season. Large black dots denote means, while error bars denote ±1*SD*. Small black dots shown to the right of the unpaired male violins represent the cuckoldry success of the four brood‐tending males in this study that engaged in cuckoldry. Different uppercase letters indicate *p* < 0.05

We applied our methods for reconstructing sire genotypes to the fry in each brood (1,976 fry from 68 territories in total) and because we had sampled nearly all the brood‐tending individuals in the quadrat each season, we could be confident in our relative assignments of fry between *brood‐tending* males and *unpaired* males. We detected 140 distinct cases of cuckoldry perpetrated by 130 different males. Of these 130 cuckolders, only four (3.1%) were identified as brood‐tending males in the sampled population (one from the dry season and three from the rainy season). When acting as cuckolders, brood‐tending males sired on average 12.8 ± 15.8 fry per cuckoldry event (*N* = 4 events), amounting to 24.3 ± 20.4% of each extra‐pair female's brood. Unpaired males, on the other hand, sired on average 5.5 ± 4.9 fry per cuckoldry event (*N* = 136 events), amounting to 17.7 ± 12.8% of each female's brood. Unfortunately, these four incidents of cuckoldry by brood‐tending males were too few to compare statistically against unpaired males. However, brood‐tending male cuckoldry success was visually comparable to that of unpaired males (see Figure 1a, b). Interestingly, fry sired by brood‐tending males in a foreign territory were always markedly different in body length (larger in three cases, smaller in one case) than fry that they had sired in their own territory (differences in body lengths between the fry ranged from 2‐fold to 2.6‐fold or 6 to 14 mm in absolute terms). This suggests a time lag of approximately 2 weeks or more between when the brood‐tending males sired fry in their own nest and in their neighbouring nests (Rossiter, 1991).

### 3.3 More fry were sired by brood‐tending males than by unpaired males

Brood‐tending males, using a combination of within‐pair and extra‐pair reproduction, sired a greater number of the genotyped fry (18.1 ± 15.1 fry per male) than unpaired males (5.5 ± 5.1 fry per male; GLM, est. ± *SE* = 1.13 ± 0.11, *z* = 9.98, *N* = 194, *p* < 0.0001, Figure 1a). The average number of fry sired by each male tactic (brood‐tending and unpaired) did not differ between the seasons (est. ± *SE* = 0.045 ± 0.11, *z* = 0.40, *N* = 194, *p* = 0.69, Figure 1a).

We also assessed the proportion of every female's brood that each male sired. Here, we detected an interaction effect indicating that the difference between brood‐tending and unpaired males, in the proportion of young that they sired, was greater in the dry season than in the rainy season (GLMM, interaction, est. ± *SE* = −1.39 ± 0.29, *t* = −4.81, *N* = 204, *p* = 0.0048, Figure 1b). This interaction was driven by the fact that brood‐tending males sired larger proportions of their broods in the dry season than the rainy season (est. ± *SE* = 1.08 ± 0.24, *t* = 4.54, *N* = 204, *p* = 0.0061, Figure 1b). Despite this interaction, brood‐tending males in both seasons sired larger proportions of their females’ broods than individual unpaired males. In the dry season, brood‐tending males sired 76.7 ± 26.5% of their females’ broods while unpaired males each sired 16.0 ± 10.4% (est. ± *SE* = 2.94 ± 0.23, *t* = 12.88, *N* = 204, *p* < 0.0001). In the rainy season, brood‐tending males sired 51.9 ± 28.6%, while unpaired males each sired 18.5 ± 13.8% (est. ± *SE* = 1.55 ± 0.19, *t* = 8.27, *N* = 204, *p* < 0.0001, Figure 1b).

### 3.4 Brood‐tending males have a higher reproductive output than unpaired males

In our “best‐case scenario” (see Methods), we estimated that individual unpaired males would have sired 10.0 ± 10.6 fry across all territories in the quadrat (13.7 ± 15.1 in the dry season and 8.1 ± 7.0 in the rainy season), representing their reproductive output over a ~100‐day time period. Note, this method likely overestimates the siring success of unpaired males, because it does not account for unpaired males that did not sire any fry. Nevertheless, the reproductive output of individual unpaired males was still lower than that of brood‐tending males over this time window (GLM, est. ± *SE* = −0.50 ± 0.11, *z* = −4.39, *N* = 194, *p* < 0.0001). Using a maximum‐likelihood approach, which estimates the number of unsampled males with zero reproductive success (see Methods), we found that unpaired males would have sired an average (±*SE*) of 2.0 ± 1.2 fry in the dry season and 1.4 ± 0.5 fry in the rainy season. Reproductive output of unpaired males may thus be substantially lower than that of brood‐tending males if unsuccessful cuckolders are accounted for.

### 3.5 Brood‐tending males are larger than unpaired males but do not have larger GSIs

**Figure 2:**
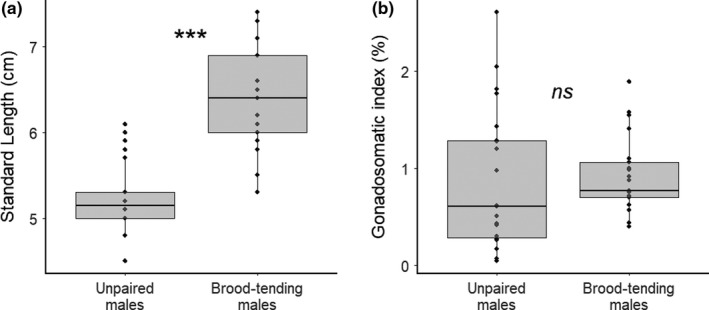
Comparison between brood‐tending pair‐bonded males and unpaired males showing that the two male types differed in (a) standard length, but not in (b) gonadosomatic index (measured as testes mass divided by eviscerated body mass). Note that these measurements were taken from fish stored in 70% ethanol for 5 days (see Methods). ***indicates *p* < 0.001

Brood‐tending males (standard length, mean ± *SD* = 6.36 ± 0.57 cm) were significantly larger than unpaired males (5.23 ± 0.42 cm; LM, est. ± *SE* = 1.13 ± 0.15, t = 7.36, *df* = 41, *p* < 0.0001, Figure 2a). After 5 days in 70% ethanol, we found that brood‐tending males had heavier testes (0.053 ± 0.026 g) in terms of absolute mass when compared to unpaired males (0.028 ± 0.025 g, est. ± *SE* = 0.025 ± 0.0077, *t* = 3.26, *df* = 41, *p* = 0.0022). However, GSI did not differ significantly between brood‐tending males and unpaired males (Wilcoxon rank sum test, *W* = 177, *p* = 0.20), though unpaired males displayed wide variance in their GSI values (Figure 2b).

## 4 DISCUSSION

The degree to which pair‐bonded males invest in within‐pair versus extra‐pair reproduction is expected to be the outcome of an evolutionary game (Fishman et al., 2003) in which males’ options—to remain on their territories or seek fertilizations elsewhere—are weighed against one another. The outcome of this game is likely dictated by numerous factors, which collectively influence the extent to which pair‐bonded males can gain extra‐pair fertilizations while also safeguarding their paternity at home. Such factors include how effective males are at guarding and/or cuckolding, the degree of breeding synchrony and infidelity among females in the population, and the mechanism and intensity of sperm competition with other males (see Kokko & Morrell, 2005). Here, we showed that brood‐tending, pair‐bonded males in a socially monogamous fish invest primarily in within‐pair reproduction and very rarely in cuckoldry.

We initially expected brood‐tending males to be highly prolific cuckolders for the following reasons. First, externally fertilizing fishes often experience high payoffs for cuckoldry tactics (see introduction). Second, socially monogamous fishes may experience a weak trade‐off between paternity assurance at home and fertilization success abroad (due to a low probability that spawnings overlap considerably in time). Indeed, parental care in *V. moorii* lasts up to ~100 days, during which time the brood‐tending mother does not spawn another brood; however, neighbouring females readily spawn in their own territories during this time (Rossiter, 1991; Aneesh P. H. Bose, Holger Zimmermann, Karoline Fritzsche, Jonathan M. Henshaw, *personal observations*). Third, the *V. moorii* territories in our study were on average 2.5 m away from one another, well within typical travel distances for adult fish. Close proximity between breeding territories is expected to facilitate extra‐pair fertilizations, as has been found in birds (Brouwer et al., 2017; Mayer & Pasinelli, 2013; Westneat & Sherman, 1997). Fourth, we also expected that in socially monogamous fishes with biparental care, such as *V. moorii*, cuckoldry by paired males would be facilitated because fry would not be left unattended in the temporary absence of the male. Brood vulnerability appears to be a major constraint on cuckoldry by territory‐holding males in some fishes with sole paternal care (e.g., three‐spined sticklebacks, *G. aculeatus*, Candolin & Vlieger, 2013; plainfin midshipman fish, *P. notatus*, Lee & Bass, 2006; Cogliati et al., 2014). Female *V. moorii* appear to be capable of caring for broods even after the experimental or natural removal of their male partners (unpublished data). Despite these seemingly favourable conditions, brood‐tending *V. moorii* males in our study rarely engaged in cuckoldry.

Could phenotypic constraints limit cuckoldry by brood‐tending males? In fishes, cuckoldry tactics generally, but not always, benefit from small body sizes (Taborsky & Brockmann, 2010). Males that rely on cuckoldry are typically smaller and often younger than those adopting conventional tactics (e.g., ocellated wrasses, *Symphodus ocellatus*, Alonzo, Taborsky, & Wirtz, 2000). We did indeed find that pair‐bonded males are larger on average than unpaired males. If cuckoldry success declines with male body size, then this may explain why brood‐tending males were relatively unsuccessful in siring extra‐pair young. Testing this idea in *V. moorii* will require a separate experiment in the future. However, it should be noted that the four brood‐tending cuckolders in our main study were not consistently smaller than the other brood‐tending males in our sample. In fact, these four males represented a span between the 20th and 100th percentiles of brood‐tending male body sizes, suggesting that large body size does not necessarily preclude cuckoldry.

Several inferences can be drawn from the four brood‐tending males that we identified as cuckolders. First, the fry that they sired in their own territories differed in body size from those that they sired in foreign territories, suggesting a time lag of approximately 2 weeks in between each spawning (estimated using offspring growth curves in Rossiter, 1991). In three cases, the extra‐pair fry were larger than the males’ within‐pair fry. There are three possible scenarios that could explain this pattern: (a) the males, while paired to their current female, cuckolded in a neighbouring nest *before* their current brood was spawned, (b) the males were *unpaired* at the time of cuckolding and since established a pair‐bond and (c) the males were originally paired with the mother of their “extra‐pair fry,” but were then ousted from their territory by one of their cuckolders, and have since established a new pair‐bond. Another inference that we can draw is from the fact that the four brood‐tending males appeared to take advantage of their extremely close proximity to their cuckolds’ territories. Indeed, they cuckolded in their nearest neighbours’ territories, which were respectively 1.4, 1.4, 0.8 and 0.6 m away. These nearest neighbour distances are shorter than the population average of 2.5 m and therefore raise the question of whether even seemingly trivial distances between territories are barriers to cuckoldry in this system. Furthermore, we used our distance measures between neighbouring nests to estimate the spatial separation between nests that were cuckolded by the *same* unpaired males. Our data set included 10 such repeat cuckolders, each having sired young in two different nests. Interestingly, eight out of these 10 cases involved cuckolding nests that were within 10 m of one another, perhaps suggesting a limited range of cuckoldry for unpaired males as well. Future studies will be required to verify these ideas with larger sample sizes.

Brood‐tending males lost ~37% of their fry to cuckoldry. This loss is high when compared to many other fish species (see Coleman & Jones, 2011; e.g., 15% loss in pumpkinseed sunfish, *Lepomis gibbosus*, Rios‐Cardenas & Webster, 2005; 28% loss in ocellated wrasses, *S. ocellatus*, Alonzo & Heckman, 2010; 37% loss in plainfin midshipman fish, *P. notatus*, Cogliati, Neff, & Balshine, 2013). We attributed the vast majority of this loss to males that had no pair‐bonds of their own and therefore would have relied solely on cuckoldry during our sampling periods. In fact, gonadosomatic indices suggest that many unpaired males in the population may also be reproductively capable (Figure 2b). Although gonadosomatic indices are a coarse measure of reproductive condition, and they assume isometry between gonadal and somatic tissues as fish grow (which may not always be true), these indices can still be informative and are widely used in fisheries biology (Zeyl, Love, & Higgs, 2014). Here, we show that some unpaired males have high gonadosomatic indices raising the possibility that a portion of the unpaired male population is indeed reproductively mature, which is consistent with our genetic paternity data. However, despite some unpaired males investing heavily into reproductive traits, and brood‐tending males losing paternity at home while not effectively offsetting these losses elsewhere, more of the genotyped fry in our study could still be assigned to brood‐tending males than to unpaired males. This was because individual unpaired males not only sired fewer fry per brood than brood‐tending males, but they also cuckolded in few territories. Of the 126 unpaired male cuckolders that we detected, only 10 sired offspring in more than one territory (and never in more than two). It is possible that unpaired males also sired fry in territories beyond the quadrat. However, because of their low rate of cuckoldry across multiple territories within the quadrat, it is unlikely that unpaired males gained considerable paternity outside the quadrat. Furthermore, we found that only five of the 44 unpaired males that were caught opportunistically during sampling had sired any fry within the quadrat, further suggesting that individual unpaired males gain few mating opportunities.

Our estimates of reproductive output suggest that brood‐tending males sire more young than unpaired males over a similar time period. Thus, despite suffering high paternity losses and not offsetting these losses with their own extra‐pair reproduction, males that secure pair‐bonds and care for offspring are still more successful than males that remain unpaired. This difference in reproductive output between the male tactics is likely driven by a relatively large pool of unpaired males in the population; collectively, unpaired males account for a high degree of paternity loss in the population, but individually, they each gain few fertilizations. Nonetheless, cuckoldry by unpaired males has the potential to reduce variance in reproductive success and hence the opportunity for sexual selection among males in this system because paired males lose paternity share to males that would otherwise not have reproduced (i.e., unpaired males) (Jones, Walker, Kvarnemo, Lindström, & Avise, 2001). A valuable next step will be to clarify whether males in this system adopt a fixed brood‐tending (i.e., conventional) or unpaired (i.e., alternative) tactic across their lives, or whether these tactics are flexible and adopted sequentially or reversibly (Taborsky & Brockmann, 2010).

We found that average brood paternity was much higher in the dry season (76.7% of offspring per brood were sired within the pair‐bond) compared to the rainy season (51.9% of offspring). While a single contrast between two field seasons is insufficient to draw any robust conclusions regarding seasonal patterns in paternity, it has been acknowledged that the success of alternative tactics can be influenced by ecological factors that vary temporally and/or spatially (Monroe, Amundsen, Utne‐Palm, & Mobley, 2016; Taborsky & Brockmann, 2010). Interestingly, even though sire number and average paternity differed between the two seasons, the *absolute* number of offspring sired by brood‐tending males did not. This suggests that (a) average brood sizes differed between the seasons, with females laying fewer eggs per brood in the dry season relative to the rainy, and/or (b) non‐kin offspring suffered higher mortality than kin offspring in the dry season relative to the rainy. It still remains to be investigated whether extra‐pair offspring experience different survival rates than within‐pair offspring in *V. moorii* (e.g., via differential allocation of parental care, offspring cannibalism or genetic differences between the offspring types) as differences in fitness‐related traits between within‐ and extra‐pair offspring have sometimes been found in other taxa (Magrath, Vedder, Van der Velde, & Komdeur, 2009).

This study is the first comprehensive examination of how brood‐tending males trade within‐pair reproduction against extra‐pair reproduction in a socially monogamous fish. The *V. moorii* mating system is characterized by considerable extra‐pair paternity, and multiple factors led us to predict that brood‐tending males would be predisposed to cuckoldry. Surprisingly, our results reveal that brood‐tending males are very rarely the cuckolders. Brood‐tending males lose paternity at home and do not effectively regain it elsewhere, though they still sired more of the genotyped fry in our study and likely enjoy a higher reproductive output than males without pair‐bonds. Which factors act to limit paired male participation in cuckoldry will be a topic for future research; possibilities include phenotypic constraints (e.g., body size limitations on cuckoldry), trade‐offs between cuckoldry and mate or brood guarding, or punishment by the social partners of unfaithful paired males. Overall, fishes display great diversity in mating systems, reproductive behaviours and parental care patterns, especially in comparison with birds and mammals. However, the multiple independent origins of social monogamy have not been exploited when investigating how pair‐bonded males handle the trade‐off between within‐pair and extra‐pair reproduction. We suggest that greater taxonomic representation, including socially monogamous fishes and other understudied taxa, may provide novel insights into this long‐standing question.

## AUTHOR CONTRIBUTIONS

K.S. and A.B. conceived and designed the study. H.Z., J.H. and K.F. conducted the field work. H.Z. and K.F. performed the microsatellite analyses. A.B., H.Z., K.F., J.H. and K.S. analysed the data. A.B. wrote the manuscript with input from all the co‐authors.

## DATA ACCESSIBILITY

Analyses reported in this article can be reproduced using the data and genotypes uploaded to DRYAD: https://doi.org/10.5061/dryad.1hs12ng.
