# Supplementary material for: Brood‐tending males in a biparental fish suffer high paternity losses but rarely cuckold
Source: Mol Ecol. 2018 Sep 27;27(21):4309–21. doi: 10.1111/mec.14857 (PMC6221093; doi:10.1111/mec.14857)
Supplement: Supplementary file 1 [file MEC-27-4309-s001.docx]

**Supplemental Information for:**

**Brood-tending males in a biparental fish suffer high**

**paternity losses but rarely cuckold**

Aneesh P H Bose, Holger Zimmermann, Jonathan M Henshaw, Karoline Fritzsche, Kristina M Sefc

1. **Outline of custom Perl script for multi-locus genotype comparisons**

Here we provide an overview of the Perl script written to compare male genotypes for the purpose of identifying pair-bonded males that acted as cuckolders and any males that cuckolded in multiple territories. This script conducted pairwise comparisons between all male genotypes (i.e. observed pair-bonded male genotypes and reconstructed cuckolder male genotypes), while implementing the following rules:

1. Alternative genotypes reconstructed for the same cuckolder male were not compared to each other. Cuckolder male genotypes could not always be unambiguously reconstructed (see Methods) and in these cases cuckolder males sometimes had several alternative genotypes. For example, when the mother and extra-pair fry were both heterozygous for the same alleles, e.g. X and Y, we scored the paternal alleles (i.e. the cuckolder male’s alleles) as X or Y, since the cuckolder could have contributed either of these two alleles. Therefore, this cuckolder male had two alternative genotypes, one containing the X allele and the other containing the Y allele. If a second ambiguity arose at a different locus, then there would be two ambiguous alleles (e.g. X or Y and A or B), and we would have generated four alternative genotypes to cover all combinations of X/Y and A/B. Out of all these alternative genotypes, one would be the true cuckolder male genotype.
2. So-called “partial genotypes” were also not compared to each other. When a cuckolder sired only a few offspring, genotype reconstructions were partial in that only one allele could be inferred at each locus. Any two such partial genotypes are always consistent with coming from a single male, rendering partial-partial genotype comparisons uninformative.
3. In order for a locus to be consistent with *identity* (i.e. the two genotypes belong to the same male), one of the following conditions had to be met:

- In comparisons between two completely genotyped loci (both alleles scored for each genotype), both alleles had to match. If this was the case, we counted two matching alleles and one matching locus. If not, we counted one mismatching locus.

- In comparisons between a completely genotyped locus (both alleles scored for this locus) and an incompletely genotyped locus (only one allele scored for this locus), the allele of the incomplete locus had to match one of the two alleles of the complete locus. If this was the case, we counted 1 matching allele and one matching locus. If not, we counted one mismatching locus.

- When both genotypes were incomplete at the locus under consideration, the locus was consistent with identity irrespective of whether the two alleles matched or not. We therefore always counted one matching locus. But if the two alleles were identical, we also counted 1 matching allele.

The number of matching alleles, matching loci, and *mis*matching loci were counted across all loci. The output reported all pairwise comparisons with the number of matching and mismatching loci, the number of matching alleles, the number of alleles scored for each genotype (the number of alleles scored sometimes varied due to partial reconstructions and missing data), and the number of alleles compared between each pair of genotypes (also variable due to missing data).

We considered two genotypes to be consistent with a single male when: 1) they were both identical, or 2) if they differed by up to two mismatching loci. This was to account for possible errors in genotyping or genotype reconstruction, though we also required at least eight matching loci. When we found a match between two such genotypes, we then considered this male to have sired offspring in both broods / territories.

1. **Estimating average reproductive success of unpaired males**

Here we derive maximum likelihood estimates for the number of unpaired males $m_{T}$ in the entire quadrat and the probability $p$ that any given male cuckolds any given territory (see Methods in main text). We assume that all males have the same propensity to cuckold, and that all territories have the same chance of being cuckolded. If we sample fry from $n_{S}$ territories, then the number of sampled territories cuckolded by any given male follows a binomial distribution $Bin(n_{S},p)$. The probability that he cuckolds exactly *k* territories is:

| $\left( \begin{matrix} n_{S} \\ k \end{matrix} \right)p^{k}\left( 1-p \right)^{n_{S}-k}$ | (S1) |
| --- | --- |

We write $M_{k}$ for the random variable representing the number of males that cuckold exactly $k$ of the sampled territories. The number of males cuckolding at least one of the sampled territories is then $M_{S}=M_{1}+M_{2}+\ldots+M_{n_{S}}$. The remaining males $M_{0}=m_{T}-M_{S}$ do not cuckold any of the sampled territories. For fixed values of the parameters $m_{T}$ and $p$, the random variables $M_{1},\ldots,M_{n_{S}}$ jointly follow a multinomial distribution, with probability mass function given by:

| $f\left( m_{1},\ldots,m_{n_{S}} \vert m_{T},p \right)=\frac{m_{T}!}{m_{0}!m_{1}!m_{2}!\cdots m_{n}!}\sum_{k=0}^{n_{S}} \left[ \left( \begin{matrix} n_{S} \\ k \end{matrix} \right)p^{k}\left( 1-p \right)^{n_{S}-k} \right]^{m_{k}}$ | (S2) |
| --- | --- |

The log-likelihood function for $(m_{T},p)$ is then:

| $\mathcal{L}\left( m_{1},\ldots,m_{n_{S}} \vert m_{T},p \right)=\log(m_{T}!)-\sum_{k=0}^{n_{S}} \log(m_{k}!)+\sum_{k=0}^{n_{S}} m_{k}\left[ \log\left( \begin{matrix} n_{S} \\ k \end{matrix} \right)+k\log p+\left( n_{S}-k \right)\log(1-p) \right]$ | (S3) |
| --- | --- |

Using the fact that $m_{0}=m_{T}-m_{S}$, the partial derivatives of the log-likelihood function with respect to $m_{T}$ and $p$ are:

| $\frac{\partial\mathcal{L}}{\partial m_{T}}=n_{S}\log(1-p)+\sum_{k=0}^{m_{S}-1} \frac{1}{m_{T}-k}$ | (S4) |
| --- | --- |

| $\frac{\partial\mathcal{L}}{\partial p}=\frac{1}{p(1-p)}\left( -pm_{T}n_{S}+\sum_{k=1}^{n_{S}} km_{k} \right)$ | (S5) |
| --- | --- |

Setting both derivatives equal to zero then yields:

| $n_{S}\log(1-p)+\sum_{k=0}^{m_{S}-1} \frac{1}{m_{T}-k}=0$ | (S6) |
| --- | --- |

| $pm_{T}=\frac{1}{n_{S}}\sum_{k=1}^{n_{S}} km_{k}$ | (S7) |
| --- | --- |

These equations can be solved numerically to obtain maximum likelihood estimates $\hat{m}_{T}$ and $\hat{p}$ given the sample values $m_{1},\ldots,m_{n_{S}}$.

We now derive approximate standard errors for these maximum likelihood estimates based on the asymptotic variance-covariance matrix. The Hessian matrix for $(m_{T},p)$ is given by:

| $H=\left( \begin{matrix} \frac{\partial^{2}\mathcal{L}}{\partial m_{T}^{2}} & \frac{\partial^{2}\mathcal{L}}{\partial m_{T}\partial p} \\ \frac{\partial^{2}\mathcal{L}}{\partial m_{T}\partial p} & \frac{\partial^{2}\mathcal{L}}{\partial p^{2}} \end{matrix} \right)=-\left( \begin{matrix} \sum_{k=0}^{m_{S}-1} \frac{1}{\left( m_{T}-k \right)^{2}} & \frac{n_{S}}{1-p} \\ \frac{n_{S}}{1-p} & \frac{1}{\left( 1-p \right)^{2}}\left( m_{T}n_{S}+\frac{1-2p}{p^{2}}\sum_{k=1}^{n_{S}} km_{k} \right) \end{matrix} \right)$ | (S8) |
| --- | --- |

We wish to find the expected value of the Hessian matrix. For the first entry $H_{11}$, we note that the probability that a particular male cuckolds at least one of the sampled territories is

$q=\left( 1-\left( 1-p \right)^{n_{S}} \right)$. The total number of sampled cuckolders $M_{S}$ thus follows a binomial distribution $Bin(m_{T},q$). Hence, we can write:

| $\mathbb{E}H_{11}=-\sum_{i=1}^{m_{T}} \left( \begin{matrix} m_{T} \\ i \end{matrix} \right)q^{i}\left( 1-q \right)^{m_{T}-i}\sum_{k=0}^{i-1} \frac{1}{\left( m_{T}-k \right)^{2}}$ | (S9) |
| --- | --- |

For the last entry $H_{22}$, we note that $\frac{1}{n_{S}}\sum_{k=1}^{n_{S}} km_{k}$ is just the average number of cuckolders per territory. Hence, we must have $\mathbb{E}\frac{1}{n_{S}}\sum_{k=1}^{n_{S}} km_{k}=pm_{T}$ and so $\mathbb{E}\sum_{k=1}^{n_{S}} km_{k}=pm_{T}n_{S}$. This means that:

| $\mathbb{E}H_{22}=-\frac{m_{T}n_{S}}{p(1-p)}$ | (S10) |
| --- | --- |

The expectations of the non-diagonal elements are straightforwardly given by:

| $\mathbb{E}H_{12}\mathbb{=E}H_{21}=-\frac{n_{S}}{1-p}$ | (S11) |
| --- | --- |

We now have everything needed to calculate the Fisher information matrix $\mathcal{I}\left( m_{T},p \right)\mathbb{=-E(}H)$. The asymptotic variance-covariance matrix of the maximum likelihood estimates is then given by $A\mathcal{=I}\left( \hat{m}_{T},\hat{p} \right)^{-1}$. We approximated the variance in the estimate $\hat{p}n_{T}\bar{f}$ of the average number of fry per unpaired male as (see Results in main text):

| $var(\hat{p}n_{T}\bar{f})=n_{T}^{2}\left( \hat{p}^{2}var(\bar{f})+\bar{f}^{2}\mathrm{var}\left( \hat{p} \right)+var(\bar{f})var\left( \hat{p} \right) \right)$ | (S12) |
| --- | --- |

This assumes that the total number of territories $n_{T}$ in the quadrat is measured with negligible error and that $\hat{p}$ is independent of $\bar{f}$.
